# *GJB2* c.235delC variant associated with autosomal recessive nonsyndromic hearing loss and auditory neuropathy spectrum disorder

**DOI:** 10.1590/1678-4685-GMB-2017-0318

**Published:** 2019-02-28

**Authors:** Hong Xia, Xiangjun Huang, Hongbo Xu, Yong-an Zhou, Lina Gong, Zhijian Yang, Jingyan Lv, Hao Deng

**Affiliations:** 1 Central South University Central South University Center for Experimental Medicine and Department of Neurology Third Xiangya Hospital ChangshaHunan China Center for Experimental Medicine and Department of Neurology, the Third Xiangya Hospital, Central South University, Changsha, Hunan, China; 2 Central South University Central South University Department of Emergency Third Xiangya Hospital ChangshaHunan China Department of Emergency, the Third Xiangya Hospital, Central South University, Changsha, Hunan, China; 3 Hunan University Hunan University Department of General Surgery First Affiliated Hospital ChangshaHunan China Department of General Surgery, the First Affiliated Hospital, Hunan University of Chinese Medicine, Changsha, Hunan, China; 4 Shaanxi Normal University Shaanxi Normal University Department of Blood Transfusion Second Affiliated Hospital TaiyuanShanxi China Department of Blood Transfusion, the Second Affiliated Hospital, Shanxi Medical University, Taiyuan, Shanxi, China

**Keywords:** Auditory neuropathy spectrum disorder, exome sequencing, hearing loss, *GJB2* gene, *GJB2* c.235delC variant

## Abstract

Autosomal recessive nonsyndromic hearing loss (ARNSHL) is a genetically heterogeneous neurosensory disorder, usually characterized by congenital or prelingual hearing loss. We report a Han Chinese male, born to consanguineous parents, presenting with nonsyndromic sensorineural hearing loss, whose clinical phenotype was also consistent with auditory neuropathy spectrum disorder (ANSD). After exome sequencing, a gap junction protein beta 2 gene (*GJB2*) c.235delC variant in the homozygous state was detected in the patient. Both parents were heterozygous for this variant, as documented by Sanger sequencing. The known pathogenic *GJB2* c.235delC variant was not detected in 200 healthy controls. It is predicted to be a disease-causing alteration by generating a truncated protein p.(L79Cfs*3), disturbing the appropriate folding and/or oligomerization of connexins and leading to defective gap junction channels. This study shows that the association of homozygosity of the *GJB2* c.235delC variant with ARNSHL and ANSD in a patient.

Autosomal recessive nonsyndromic hearing loss (ARNSHL) is a genetically heterogeneous neurosensory disorder, usually characterized by congenital or prelingual hearing loss, and not accompanied by other clinical features (Xia *et al.*, 2015; Meena and Ayub, 2017). ARNSHL accounts for 45-52.5% of cases of inherited hearing loss, which occurs in approximately 1/1000-2000 newborns (Duman and Tekin, 2012; Duan *et al.*, 2015; Xia *et al.*, 2015). Individuals with ARNSHL usually present difficulty in language development and social interactions.

Since variants in the gap junction protein beta 2 gene (*GJB2*) were first identified as causative of ARNSHL in 1997 (Kelsell *et al.*, 1997), to date (January, 2019), at least pathogenic variants in other 72 genes have been causally associated with ARNSHL according to the Hereditary Hearing Loss Homepage (https://hereditaryhearingloss.org).

The extreme genetic heterogeneity of nonsyndromic hearing loss makes the use of regular Sanger sequencing to identify its genetic cause very challenging, and exome sequencing has been recommended as a cost-effective alternative strategy (Xia *et al.*, 2016). In the present study, homozygosity for a *GJB2* variant was detected by exome sequencing, as causative of autosomal recessive hearing loss in a Han Chinese male presenting auditory neuropathy spectrum disorder (ANSD).

Three members of a Han Chinese family from Hunan, including two normal-hearing first-cousin parents (III:1 and III:2, Figure 1A) and a patient (IV:1, a 27-year-old male), took part in this study. Bilateral prelingual hearing impairment was diagnosed in his first year of life, but neither hearing aids nor cochlear implantation was offered during his childhood. Two hundred unrelated subjects (female/male: 100/100, aged 27.0 ± 6.8 years) without hearing impairments were recruited as healthy controls. Clinical and audiological evaluations were performed on all participants at the Third Xiangya Hospital of Central South University, Changsha, China. Peripheral blood samples were obtained from all participants, and genomic DNA was extracted using a saturated phenol-chloroform extraction method (Yuan *et al.*, 2015). The present study was reviewed and approved by the Institutional Review Board of the Third Xiangya Hospital, Central South University (Changsha, China), in accordance with the Declaration of Helsinki. Written informed consent forms were provided by all participants.

**Figure 1:**
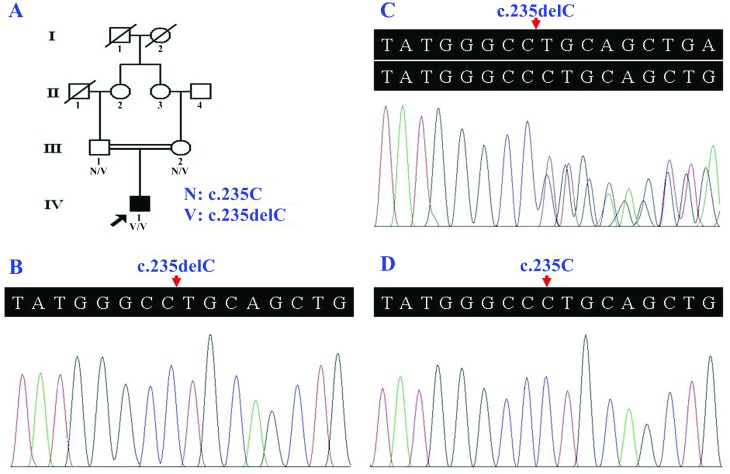
Pedigree of the Han Chinese individual with nonsyndromic hearing loss, and the *GJB2* Sanger sequencing electropherograms. (A) The patient was born to first-cousin normal-hearing parents. (B) Homozygosity for the *GJB2* c.235delC variant in the individual with hearing loss (IV:1). (C) The heterozygosity for the *GJB2* c.235delC variant in the normal-hearing father (III:1). (D) The *GJB2* sequence in a normal control.

A series of auditory evaluations, including pure tone audiometry (PTA), tympanometry, acoustic reflex (AR) thresholds, auditory brainstem responses (ABR), transient evoked otoacoustic emission (TEOAE), and distortion product otoacoustic emission (DPOAE) were performed. Potential inner ear congenital malformations were evaluated with magnetic resonance imaging (MRI). Audiometric thresholds were evaluated at frequencies 250, 500, 1000, 2000, 4000, and 8000 Hz by PTA. Hearing acuity is considered ‘normal’ at a threshold within 25 decibels (dB), and the degree of hearing loss was classed as mild (26-40 dB), moderate (41-60 dB), severe (61-80 dB), or profound (> 81 dB) (Asghari *et al.*, 2017). TEOAE and DPOAE were tested using GN otometrics-Madsen capella^TM^. Fast-Screen mode and 80 dB hearing level were set for TEOAE examination. DP1, 65 dB hearing level for f1, and 55 dB hearing level for f2 were set for DPOAE.

Three micrograms of the patient’s genomic DNA was used for exome sequencing. It was first sonically sheared and then enriched, hybridized, and captured by the Agilent SureSelect Human All Exon V4 kit at BGI-Shenzhen (Shenzhen, China), according to the manufacturer’s protocol. The library with the targeted exome was sequenced using the Illumina HiSeq 2000 platform. The mean sequencing depth was 101.78, and 99.43% of the targeted exome was covered. Single nucleotide polymorphisms (SNPs) and insertions/deletions (InDels) were detected. Alleles with a frequency > 0.5% in the following databases were screened out based on the SNPs database (dbSNP version 138), 1000 genomes project (1000 genomes release phase 3), HapMap project (2010-08_phase II + III), Exome Sequencing Project 6500 (ESP6500) (Zheng *et al.*, 2016), Exome Aggregation Consortium, and an in-house exome database of BGI. The functional effects of non-synonymous SNPs in the coding regions were predicted by Sorting Intolerant from Tolerant (SIFT, http://sift.jcvi.org/) and Polymorphism Phenotyping version 2 (PolyPhen-2, http://genetics.bwh.harvard.edu/pph2/).

Sanger sequencing was performed to identify whether candidate variants co-segregated with ARNSHL phenotype in the family, using an ABI3500 sequencer (Applied Biosystems, Foster City, CA, USA) (Zheng *et al.*, 2016). Primer sequences for the pathogenic variant in the *GJB2* gene were designed and synthesized as follows: forward, 5’-TCGCATTATGATCCTCGTTG-3’ and reverse, 5’-CTCCCCCTTGATGAACTTCC-3’. The function effects of possible candidate variants were further predicted using MutationTaster (http://www.mutationtaster.org/).

The patient’s audiological evaluation revealed profound bilateral sensorineural hearing loss, a type A tympanometric curve, and absent AR and ABR. TEOAE and DPOAE were absent in the patient’s left ear. TEOAE and low amplitude DPOAE at 4000 or 8000 Hz were elicited in the patient’s right ear. MRI showed no anomaly in the patient’s inner ears. The patient’s clinical phenotype was also consistent with ANSD, a disorder of the auditory pathway characterized by the absence of ABR and the presence of OAE (Manchaiah *et al.*, 2011). PTA of his parents showed normal hearing level.

Exome sequencing generated 104,662 SNPs and 16,813 InDels. After screening out common and nonpathogenic variants, homozygosity for the c.235delC variant (rs80338943, a known pathogenic variant, NM_004004.5) in the *GJB2* gene was found, and there were no other potentially causative variants for hearing loss.

Homozygosity for the c.235delC variant in the *GJB2* gene was confirmed in the patient by Sanger sequencing (Figure 1B). His parents were found to be heterozygous for this variant (Figure 1C). The *GJB2* c.235delC variant was not detected in the 200 healthy controls (Figure 1D), and it is predicted to be disease-causing by MutationTaster, resulting in a shift in the reading frame at codon 79 and a premature stop codon at codon 81, p.(L79Cfs*3).

Variants in the *GJB2* gene are the primary cause of ARNSHL and responsible for 5-43% of nonsyndromic hearing loss in different ethnicities (Kenneson *et al.*, 2002; Duan *et al.*, 2015). Presently at least 400 pathogenic variants in the *GJB2* gene are known on the basis of the Human Gene Mutation Database (http://www.hgmd.cf.ac.uk/ac/index.php). Mutation spectrum and frequency in the *GJB2* gene vary with ethnicity (Zheng *et al.*, 2015).

In this study, by exome sequencing, a homozygous *GJB2* c.235delC variant, known to be pathogenic (Dai *et al.*, 2015), was found in an individual with hearing loss, inherited from first-cousin normal-hearing heterozygous parents. Variants in other causative genes for hearing loss were excluded. Exome sequencing is a powerful strategy for accurate diagnosis of ARNSHL or ANSD, a highly genetically heterogeneous disorder.

The *GJB2* gene encodes connexin 26, a gap-junction protein, expressed in the human and rat cochlear cells (Kelsell *et al.*, 1997). Connexin 26 consists of an N-terminal helix, four transmembrane helices (TM1-4), two extracellular loops (E1 and E2), a cytoplasmic loop (CL), and a C-terminus (Maeda *et al.*, 2009). The protein is involved in recycling potassium ions (Kelsell *et al.*, 1997), ATP release, intercellular signaling, hearing function regulation (Zhao *et al.*, 2005), cochlear development, and active cochlear amplification (Chen *et al.*, 2014). Connexin 26 knockout mice displayed congenital hearing loss and cochlear developmental disorders (Chen *et al.*, 2014). Conditional knockout mice showed severe hearing loss and DPOAE reductions (Zhu *et al.*, 2013).

The c.235delC variant in the *GJB2* gene, predicted to produce a truncated protein, was reported in different populations, especially in East Asia (Dai *et al.*, 2015; Taniguchi *et al.*, 2015). The *GJB2* c.235delC variant involving the TM2 domain is predicted to be a disease-causing alteration by MutationTaster. It generates a truncated protein p.(L79Cfs*3) missing important functional segments, including CL, TM3, E2, TM4, and C-terminal segments. The glutamine (p.Q80) in the TM2 segment of connexin 26 interacts with arginine (p.R32) in the TM1 segment, thus the variant may interfere with the interplay between the two TM domains, disturb the appropriate folding and/or oligomerization of connexins, and lead to defective gap junction channels (Maeda *et al.*, 2009).

*GJB2* variants have been reported in 7.5% of patients with ANSD (Carvalho *et al.*, 2016). Our patient was diagnosed with ANSD due to the presence of right ear OAE, but the absence of ABR, and this is the first report of the *GJB2* c.235delC variant in connection with ANSD. ANSD may result from an abnormality in the inner hair cells (IHC), in the synapse between IHC and auditory nerve, or in the auditory nerve itself (Starr *et al.*, 1996). Connexin 26 is expressed in the cochlear basement membrane on which the hair cells lie (Kelsell *et al.*, 1997). Connexin 26 expression contributes to IHC functional maturation (Johnson *et al.*, 2017), thus *GJB2*-associated ANSD may be caused by immature IHCs.

In conclusion, our study shows a novel association of homozygosity for the c.235delC variant in the *GJB2* gene with the phenotypes of ARNSHL and ANSD.
